# Molecularly confirmed neoscytalidium dimidiatum onychomycosis in an immunocompetent Iranian woman: a case report and comprehensive literature review

**DOI:** 10.1016/j.idcr.2026.e02812

**Published:** 2026-10-22

**Authors:** Fuad Haghighat, Seyed Jamal Hashemi, Shahram Mahmoudi, Abbas Rahimi Foroushani, Kimia Kamali Sarvestani, Mohsen Gerami Shoar, Roshanak Daie Ghazvini, Leila Hossein pour, Mahsa Naeimi Eshkaleti, Hasti Kamali Sarvestani

**Affiliations:** a Department of Medical Parasitology and Mycology, School of Public Health, Tehran University of Medical Sciences, Tehran, Iran; b Department of Parasitology and Mycology, School of Medicine, Iran University of Medical Sciences, Tehran, Iran; c Department of Epidemiology and Biostatistics, School of Public Health, Tehran University of Medical Sciences, Tehran, Iran; d Department of Molecular and Cellular Biology, Zand Institute of Higher Education, Shiraz, Iran; e Department of Medical Parasitology and Mycology, Faculty of Medicine, Tehran Medical Sciences, Islamic Azad University, Tehran, Iran

**Keywords:** Onychomycosis, Neoscytalidium dimidiatum, Non-dermatophyte mold, Dematiaceous fungi, ITS sequencing, Molecular diagnosis, Nail infection

## Abstract

**Background:**

Onychomycosis is one of the most common fungal infections of the nails and is predominantly caused by dermatophytes. However, non-dermatophyte molds (NDMs), including dematiaceous fungi, are increasingly recognized as clinically relevant pathogens. *Neoscytalidium dimidiatum* is an uncommon cause of onychomycosis, reported predominantly in tropical and subtropical regions, although cases from temperate settings have also been described.

**Case presentation:**

We report a case of distal and lateral subungual onychomycosis (DLSO) caused by *N. dimidiatum* in a 54-year-old immunocompetent Iranian woman without underlying systemic disease or immunosuppressive therapy. She presented with progressive discoloration and dystrophic changes of a toenail following previous trauma. Direct microscopy of nail specimens using potassium hydroxide (KOH) demonstrated septate, branching fungal hyphae with occasional pigmentation. Culture on Sabouraud dextrose agar yielded a rapidly growing melanized-mold with characteristic dark pigmentation. Given the phenotypic overlap among *Neoscytalidium* species, molecular identification was performed. Internal transcribed spacer (ITS) rDNA sequencing identified the isolate as *N. dimidiatum*, with more than 99% sequence identity to reference sequences. The patient received combination therapy with oral itraconazole, amorolfine 5% nail lacquer, and topical sertaconazole 2%, with marked clinical improvement after 3 months; mycological cure remained under evaluation.

**Conclusion:**

This case contributes to the limited literature on *N. dimidiatum* onychomycosis by documenting molecularly confirmed DLSO in an immunocompetent Iranian woman and demonstrating concordant clinical, mycological, and molecular findings. It highlights the diagnostic challenges of NDM onychomycosis and supports an integrated approach combining direct microscopy, culture, and molecular identification for accurate species-level diagnosis and appropriate clinical management.

## Introduction

Onychomycosis is the most common fungal infection of the nail and accounts for approximately half of all nail disorders. The disease is predominantly caused by dermatophytes; however, yeasts and non-dermatophyte molds (NDMs) are increasingly recognized as clinically relevant etiological agents. Distal and lateral subungual onychomycosis (DLSO) is the predominant clinical pattern and is characterized by progressive nail discoloration, subungual hyperkeratosis, onycholysis, and nail dystrophy. Although generally considered a localized infection, onychomycosis may substantially impair quality of life and can be particularly difficult to diagnose and treat when caused by NDMs [1], [2], [3].

Among NDMs, melanized molds of the genus *Neoscytalidium* have emerged as uncommon but clinically important causes of superficial fungal infections. *Neoscytalidium dimidiatum*, formerly classified as *Scytalidium dimidiatum*, is a melanized filamentous fungus belonging to the family Botryosphaeriaceae. It has been increasingly recognized as a cause of onychomycosis and dermatomycosis, particularly in tropical and subtropical regions. Although most infections are superficial, *N. dimidiatum* has also been associated with subcutaneous and invasive infections, particularly in immunocompromised individuals [4], [5], [6].

Diagnosis of *N. dimidiatum* infection can be challenging. Its phenotypic characteristics may overlap with those of closely related species, including *Neoscytalidium novaehollandiae*, and morphological features may vary depending on culture conditions and the stage of fungal growth. In addition, the interpretation of NDMs isolated from nail specimens requires careful correlation with clinical findings and direct microscopy because these organisms may represent colonization or contamination rather than true infection. Molecular approaches, particularly sequencing of the internal transcribed spacer (ITS) region of ribosomal DNA, can therefore provide reliable species-level identification and are especially valuable when conventional phenotypic identification is inconclusive [7], [8], [9]. Accordingly, the clinical significance of an NDM isolate should be established by integrating clinical findings with direct microscopy, culture, repeat isolation or histopathological evidence when available, rather than relying on culture or molecular identification alone.

The treatment of *N. dimidiatum*-associated onychomycosis remains challenging because of variable antifungal susceptibility, limited clinical evidence, and the absence of organism-specific therapeutic guidelines. Published cases have reported inconsistent responses to both systemic and topical antifungal agents, and combination treatment has been used in some recalcitrant infections. Consequently, accurate identification of the causative organism is important for appropriate clinical management and for distinguishing true NDM onychomycosis from incidental isolation [10], [11], [12].

Herein, we report a case of distal and lateral subungual onychomycosis caused by molecularly confirmed *N. dimidiatum* in an immunocompetent Iranian woman. The present case adds to the limited clinical literature on *N. dimidiatum*-associated nail infection by providing detailed clinical, mycological, and molecular characterization and describing the therapeutic approach and early clinical response. The case is further discussed in the context of previously published reports regarding the clinical spectrum, geographic distribution, diagnostic methods, treatment strategies, and outcomes of *Neoscytalidium*-associated nail infections.

## Case Presentation

A 54-year-old Iranian woman was referred to the Department of Medical Mycology, School of Public Health, Tehran University of Medical Sciences, Tehran, Iran, for evaluation of progressive discoloration and dystrophic changes involving a toenail. She had no known underlying systemic disease and was not receiving immunosuppressive therapy. Her medical history was notable for a traumatic injury to the affected toenail approximately 12 months before presentation. According to the patient, nail discoloration and distal dystrophic changes had developed approximately 3 months before referral and had gradually progressed.

Physical examination revealed yellow-brown discoloration, distal onycholysis, and subungual hyperkeratosis of the affected toenail. The proximal nail fold and surrounding periungual skin were unremarkable, with no evidence of paronychia or cutaneous fungal infection. The interdigital spaces and the remaining fingernails and toenails appeared clinically normal. Based on the clinical findings, the lesion was classified as distal and lateral subungual onychomycosis (DLSO) (Fig. 1).

**Fig. 1:**
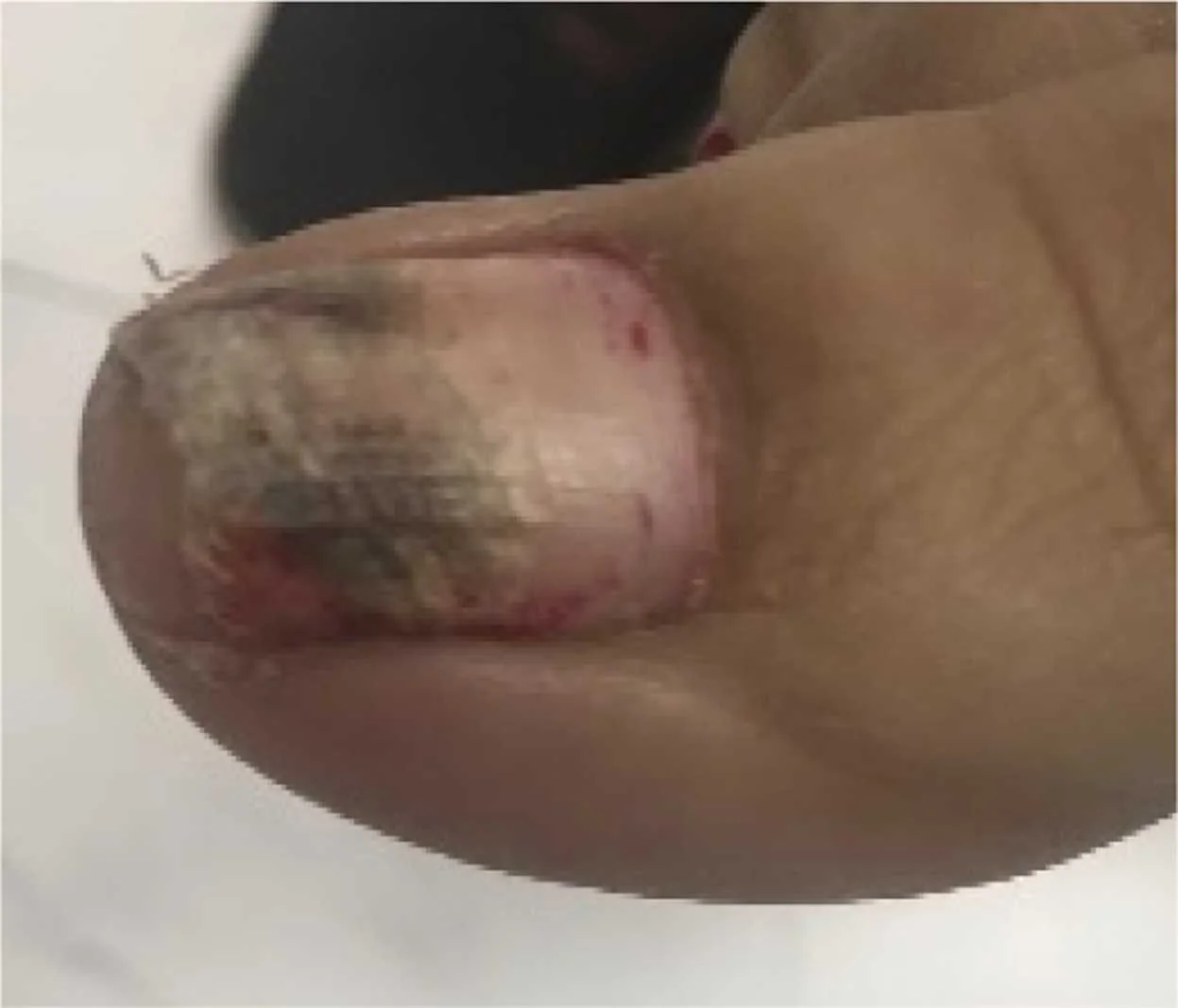
The toenail clinical presentation of DLSO caused by *N. dimidiatum*.

Nail scrapings and clippings were obtained from the distal affected area after cleansing the nail surface with 70% alcohol. To further assess the clinical significance of the NDM isolate and minimize the possibility of contamination, repeat nail sampling was performed on a separate occasion. Direct microscopic examination using a 20% potassium hydroxide (KOH) preparation demonstrated septate, branching fungal hyphae with occasional pigmentation, supporting the presence of fungal elements within the affected nail (Fig. 2). The specimen was subsequently cultured on Sabouraud dextrose agar (SDA; BD Diagnostics, Franklin Lakes, NJ, USA) and incubated at 25°C. Rapid fungal growths was observed within 7 days, with progressive darkening of the colony surface beginning at approximately 4 days of incubation. No dermatophyte was recovered from the cultured nail specimen. This finding, together with positive direct microscopy and recovery of the same melanized mold compatible with *N. dimidiatum*, supported the interpretation of the isolate as clinically significant rather than an incidental contaminant (Fig. 3 A).

**Fig. 2:**
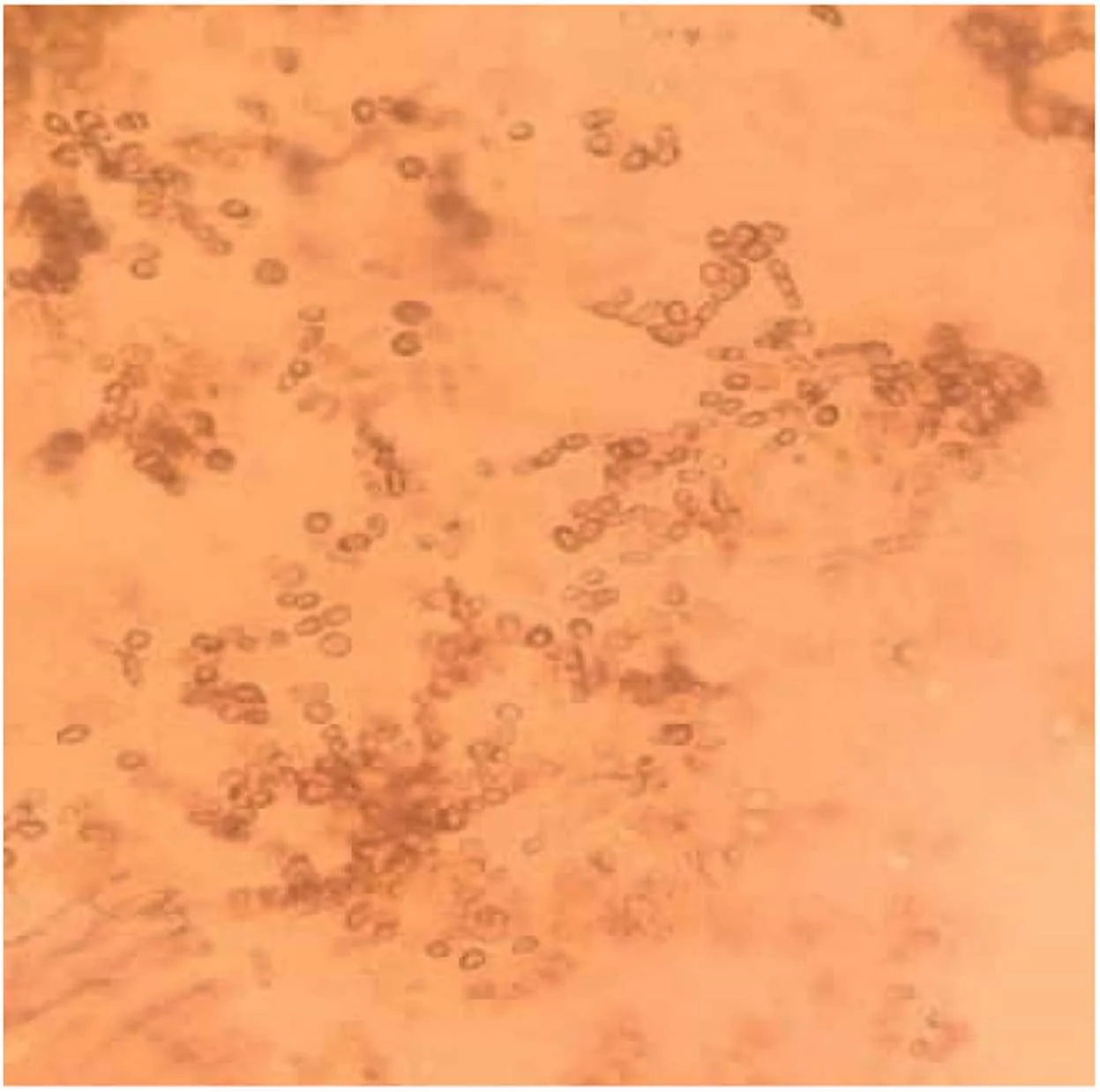
Direct microscopic examination of toenail scraping in 20% KOH, microscopic magnification was 400x.

**Fig. 3:**
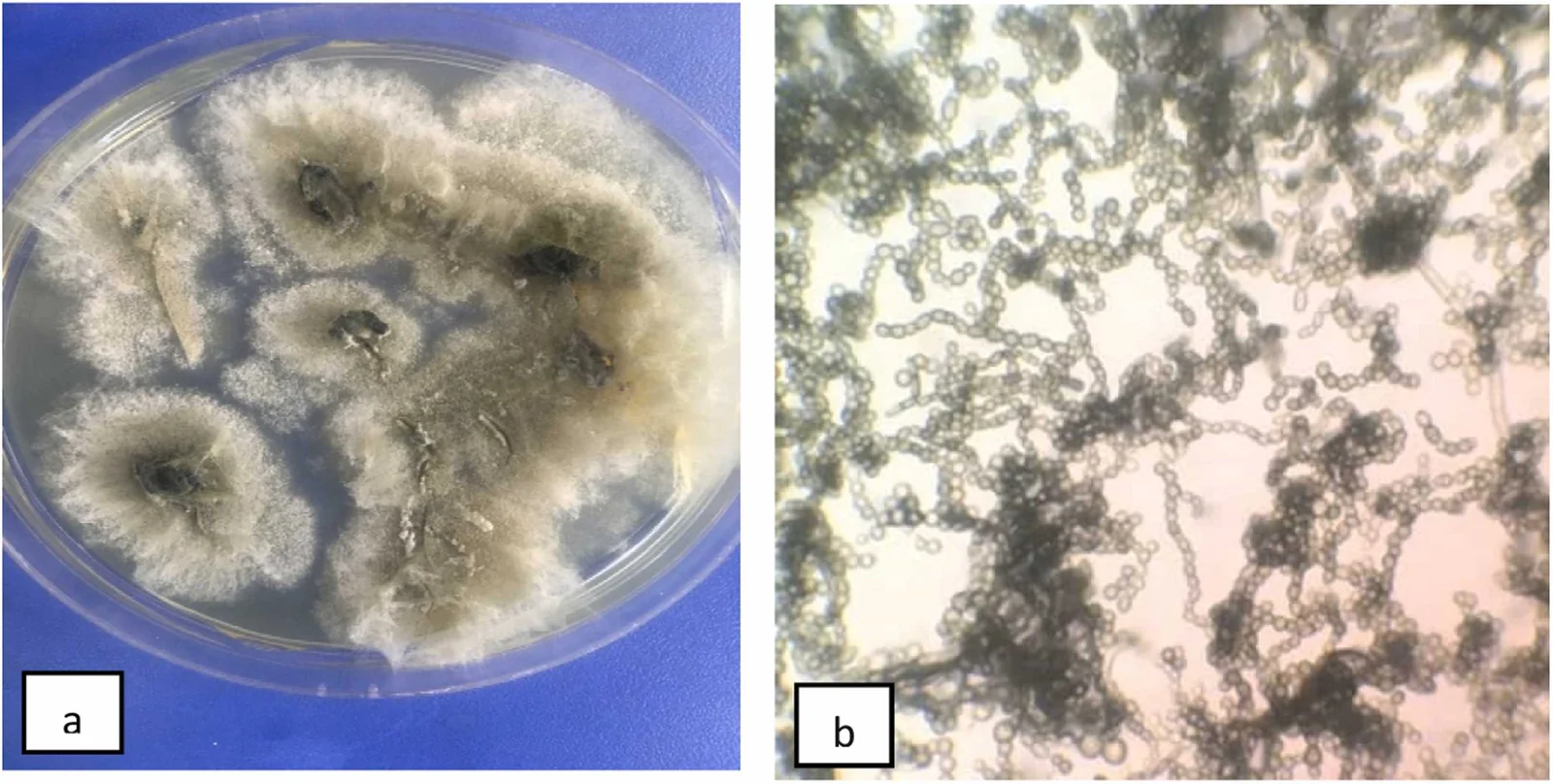
a; Macroscopic and b; microscopic appearance of *N. dimidiatum* isolated of toenail, microscopic magnification was 400x. Staining LPCB.

Microscopic examination of the cultured isolate using lactophenol cotton blue (LPCB) staining revealed septate, branching hyphae with features suggestive of a melanized or dematiaceous filamentous mold (Fig. 3B). Although the combination of colony morphology and microscopic characteristics raised suspicion for *Neoscytalidium*, phenotypic examination alone was considered insufficient for reliable species-level identification. Therefore, molecular confirmation was performed.

Genomic DNA was extracted from fresh fungal colonies using the phenol-chloroform method. The internal transcribed spacer (ITS) region of the fungal ribosomal DNA was amplified using the universal fungal primers ITS1 (5′-CTTGGTCATTTAGAGGAAGTAA-3′) and ITS4 (5′-TCCTCCGCTTATTGATATGC-3′). The amplified ITS product was subjected to sequencing, and the resulting sequence was analyzed using the NCBI BLAST database. The isolate demonstrated more than 99% sequence identity with reference sequences of *Neoscytalidium dimidiatum*. The obtained sequence was deposited in GenBank under accession number PX653220. ¹

Based on the concordance of the clinical presentation, direct microscopy, culture characteristics, microscopic morphology, and molecular identification, a diagnosis of DLSO caused by *N. dimidiatum* was established.

The patient was treated with oral itraconazole at a dose of 200 mg/day in combination with amorolfine 5% nail lacquer applied twice weekly and topical sertaconazole 2% applied twice daily. At the 3-month follow-up visit, marked clinical improvement was observed, with a reduction in nail discoloration and dystrophic changes. Treatment was continued at the time of this report, and further follow-up was planned to assess sustained clinical improvement and mycological clearance. The chronological sequence of clinical presentation, diagnostic evaluation, molecular identification, treatment, and follow-up is summarized in Fig. 4.

**Fig. 4:**
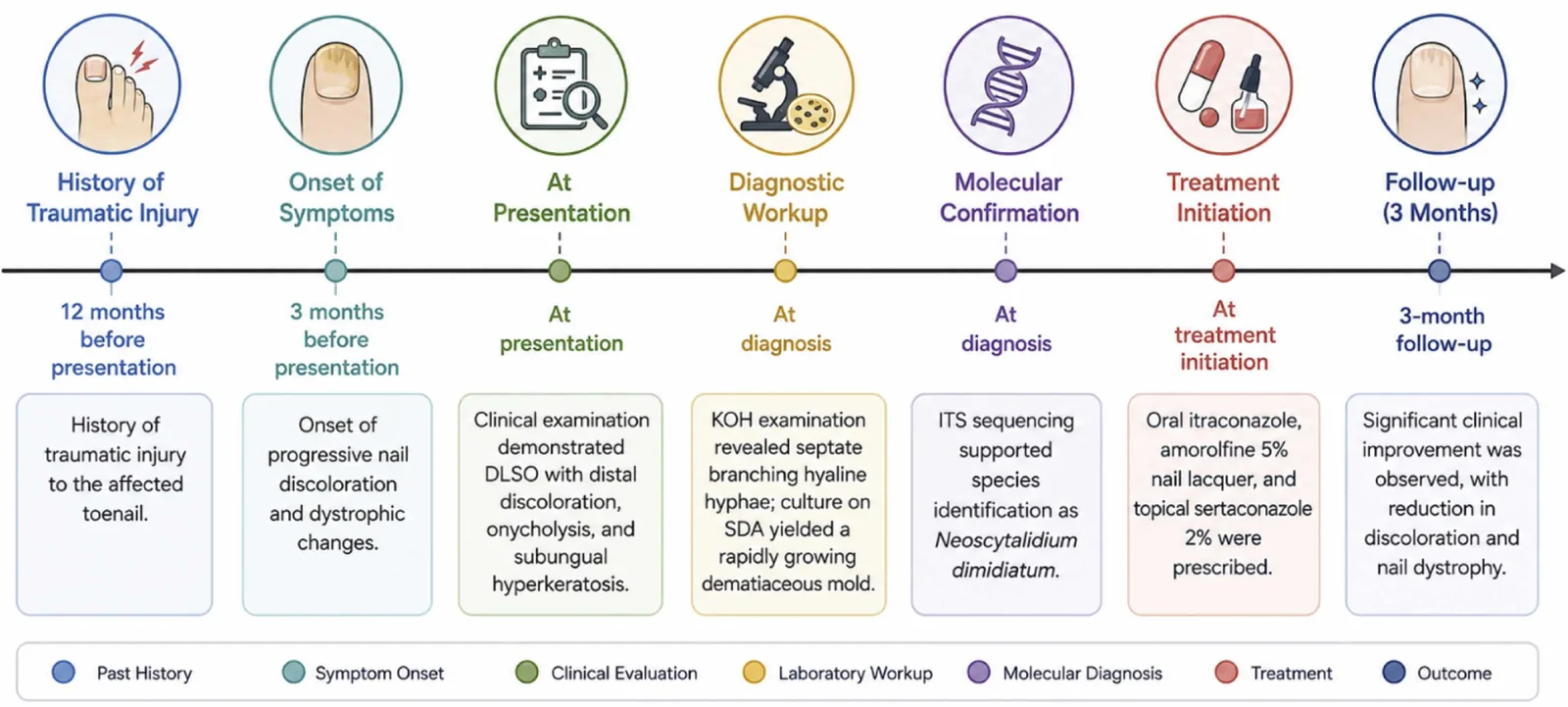
Timeline of the clinical presentation, diagnostic evaluation, molecular confirmation, treatment, and 3-month follow-up of traumatic onychomycosis caused by *Neoscytalidium dimidiatum*. KOH microscopy and culture supported the diagnosis, which was confirmed by ITS sequencing. Clinical improvement was observed after antifungal therapy.

## Mycological and Molecular Investigations

The diagnosis was supported by concordant direct microscopic, cultural, morphological, and molecular findings. Direct KOH examination demonstrated septate, branching fungal hyphae within the nail specimen. Culture on SDA yielded a rapidly growing melanized mold with progressive dark pigmentation during incubation. LPCB examination demonstrated septate, branching hyphae compatible with a dematiaceous filamentous fungus.

Because these phenotypic characteristics are not sufficient to reliably discriminate among closely related melanized molds, molecular identification was performed. Amplification and sequencing of the ITS region of rDNA using the universal fungal primers ITS1 and ITS4 demonstrated more than 99% sequence identity with reference sequences of *N. dimidiatum* in the NCBI BLAST database. The molecular findings corroborated the clinical and conventional mycological observations and established species-level identification of the isolate as *N. dimidiatum*.

## Search Strategy and Selection Criteria

A structured literature search was conducted to identify published reports of human nail infection caused by *Neoscytalidium* species and their historical taxonomic synonyms, with the aim of placing the present case within the existing clinical literature. PubMed/MEDLINE, Scopus, Web of Science, and Google Scholar were searched from database inception through June 2025. No date or language restrictions were applied during the initial search.

The search strategy included combinations of the terms "*Neoscytalidium dimidiatum*," "*Neoscytalidium novaehollandiae*," "*Scytalidium dimidiatum*," "*Scytalidium hyalinum*," "*Hendersonula toruloidea*," and "*Nattrassia mangiferae*" with terms including "onychomycosis," "nail infection," "dermatomycosis," "non-dermatophyte mold," and "case report," using the Boolean operators AND and OR. Reference lists of all retrieved articles and relevant previous reviews were also manually screened to identify additional eligible publications not captured by the electronic search.

Studies were eligible if they described individual cases, case series, or clinical or epidemiological studies documenting human ungual infection attributed to a *Neoscytalidium* species under a current or historical taxonomic designation. Reports in which nail involvement occurred concomitantly with cutaneous infection were also eligible, provided that the nail infection was clearly documented. Mycological confirmation by direct microscopy and/or culture was required; molecular identification was not mandatory. Non-English-language articles for which no accessible translation was available, conference abstracts lacking sufficient clinical or mycological information, reports limited to environmental or non-human isolates, and duplicate publications were excluded. Titles and abstracts were initially screened for relevance, followed by full-text assessment of potentially eligible studies.

For each included study, the following information was extracted: first author and year of publication, country of origin, patient demographics, clinical presentation and affected nail(s), clinical pattern of onychomycosis, identified *Neoscytalidium* species or historical taxonomic designation, diagnostic methods, treatment administered, study design, and reported clinical and/or mycological outcome. Historical nomenclature was recorded and subsequently standardized where appropriate to facilitate comparison across publications.

The search identified 42 eligible published studies addressing *Neoscytalidium* or historically related *Scytalidium* nail infections between 1979 and 2025. Their principal clinical, diagnostic, therapeutic, and outcome characteristics are summarized in Table 1 and are further discussed in relation to the present case.

**Table 1:** Published cases and epidemiological studies of *Neoscytalidium* and *Scytalidium* nail infection.

Reference / year	Country	Clinical presentation / site (n)	Treatment	Diagnosis	Diagnostic methods	Study design	Clinical / mycological outcome
Peiris et al., 1979 [13]	UK; patients from West Indies	5 men; fingernails/toenails with palmoplantar and interdigital involvement; mixed infection in 2 cases	Griseofulvin 750 mg/day × 3 yr (case 1); topical clotrimazole/miconazole under occlusion (cases 2–5)	*S. hyalinum*; mixed with *T. mentagrophytes* or *N. dimidiatum*	KOH 30%; SDA culture ± cycloheximide; microscopy; griseofulvin MIC	Case series	Limited clinical response; persistent positive cultures
Carruthers et al., 1982 [14]	Canada; patient from Fiji	35 M; fingernails, toenails, plantar scaling	Griseofulvin 1 g/day + topical haloprogin	*N. dimidiatum*	KOH 10%; SDA without cycloheximide; repeated cultures	Case report	Skin improved slightly; nail disease persisted
Gugnani et al., 1986 [15]	Nigeria	20 M; fingernails	Griseofulvin 1 g/day × 2 wk; then topical clotrimazole	*N. dimidiatum*	KOH; SDA + chloramphenicol; hair perforation; susceptibility testing	Case report	Griseofulvin-resistant; favorable response to clotrimazole
Rollman, et al., 1987 [16]	Sweden; patient from Nigeria	30 M; fingernails; associated palmoplantar disease	Griseofulvin × 1 yr (failed); nail avulsion/curettage + ciclopiroxolamine	*N. dimidiatum*	KOH; SDA without cycloheximide; PAS histology	Case report	Nail onychomycosis successfully treated; skin disease persisted
Summerbell et al., 1989 [17]	Canada	22 nail infections: 19 *N. dimidiatum*, 3 *S. hyalinum*	NR	*N. dimidiatum* / *S. hyalinum*	KOH 25% + glycerol; culture on Littman oxgall and SDA	Retrospective survey, 1986–1989	Epidemiological characterization; treatment not assessed
Kane et al., 1990 [18]	Canada	76 F; toenail	10% boric acid in mineral oil BID × 3 mo	*N. dimidiatum*	KOH 25% + glycerol; SDA ± cycloheximide	Case report	Clinical resolution after treatment
Maslen et al., 1992 [19]	Australia	74 M; toenail	No treatment	*S. hyalinum*	KOH; SDA/DTM culture; reference strain deposition	Case report	First Australian clinical isolation; treatment not assessed
Little and Hammond, 1995 [20]	Australia	6 patients; mainly toenails; palmoplantar disease in 1 case	Griseofulvin in case 1; others NR	*N. dimidiatum*	Direct microscopy; SDA without cycloheximide	Case series	Limited treatment information
Elewski, 1996[21]	USA	72 M; all toenails + 2 fingernails	Griseofulvin × 2; fluconazole 300–400 mg/week	*N. dimidiatum*	KOH; culture; nail histopathology; subsequent species identification	Case report	Refractory disease; resistance to multiple systemic agents
Lacaz et al., 1999 [22]	Brazil	2 cases: fingernail (1), toenail (1)	Itraconazole; ketoconazole + topical iodine	*N. dimidiatum*	KOH 20%; culture on potato agar	Case series	Both patients lost to follow-up
Arrese et al., 2001 [23]	Belgium; patients from Morocco	4 M; toenails; shared barefoot mosque exposure	Fluconazole, itraconazole, or terbinafine × 3 mo	*N. dimidiatum*	Histology; direct microscopy; SDA culture; repeat cultures	Case series	No mycological cure; cultures remained positive
Lacroix et al., 2003 [24]	France	332 patients; 68 toenail and 2 fingernail cases	NR	*S. hyalinum* (178), *N. dimidiatum* (146), mixed (8)	Direct microscopy; SDA without cycloheximide; macro-/micromorphology	Retrospective epidemiological study, 1994–1999	Epidemiological characterization
Alvarez et al., 2004 [25]	Colombia	183 confirmed onychomycosis; *S. dimidiatum* in 46 nail cases; mixed infections reported	NR	*N. dimidiatum*	KOH 20% + Parker stain; SDA + antibiotics; macro-/micromorphology	Cross-sectional study	Epidemiological characterization
Pontarelli et al., 2005 [2]	Brazil	2 patients; toenails	Itraconazole; fluconazole + topical amorolfine	*N. dimidiatum*	KOH 20%; SDA culture	Case report	Outcome not reported
Barua et al., 2007 [2]	India	2 F; thumbnails; green tea leaf pluckers	NR	*N. dimidiatum*	KOH 20%; SDA; LPCB; culture morphology	Case series	Occupationally associated nail infection
Alvarez and Caicedo, 2007 [26]	Colombia	1/504 university students; great toenail	NR	*N. dimidiatum*	KOH 20%; selective agar and SDA	Cross-sectional screening study, 2001–2003	Low prevalence in screened population
Xavier et al., 2010 [27]	Brazil	54/6173 nail samples; fingernails/toenails	NR	*S. dimidiatum* (74.3%), *S. hyalinum* (25.7%)	KOH 20%; Sabouraud culture	Retrospective cross-sectional study	*Scytalidium* spp. prevalence: 0.87%
Simonnet et al., 2011 [28]	French Guiana	29 toenail cases; 205 total onychomycosis patients	NR	*N. dimidiatum*	KOH 20%; culture ± cycloheximide; morphology	Retrospective cross-sectional study	Epidemiological characterization
Cursi et al., 2011 [11]	Brazil	30/1295 patients; toenails	Previous topical/systemic treatment in 63.3%; details NR	*Neoscytalidium* spp.	KOH; Sabouraud culture; macro-/micromorphology	Cross-sectional observational study	Treatment outcome not reported
Spriet et al., 2012 [29]	Belgium; patient from Nigeria	30 F; fingernail with palmoplantar disease	Terbinafine, itraconazole, miconazole, amorolfine (failed); nail extraction + voriconazole 200 mg BID × 12 wk	*N. dimidiatum*	SDA culture; CLSI/Etest susceptibility testing	Case report	Clinical and mycological response to voriconazole
Nkondjo Minkoumou et al., 2012 [30]	Cameroon	2 *N. dimidiatum* cases among 52 confirmed onychomycoses; fingernail ± toenail	NR	*N. dimidiatum*	KOH 20%; Sabouraud ± cycloheximide; macro-/micromorphology	Cross-sectional epidemiological study	Epidemiological characterization
Vasconcellos et al., 2013 [31]	Brazil	18 confirmed onychomycoses; 1 *Neoscytalidium* case involving toenails	NR	*Neoscytalidium* sp.	KOH; SDA + chloramphenicol/Mycosel	Cross-sectional observational study	Epidemiological characterization
Morales-Cardona et al., 2014 [32]	Colombia	317 onychomycosis cases; *N. dimidiatum* in 178 mould-positive cases; mainly great toenails	NR	*N. dimidiatum*	KOH 40%; SDA + antibiotics; ≥ 2 consecutive positive cultures; morphology	Retrospective cross-sectional study	*N. dimidiatum* was the predominant mould
Bunyaratavej et al., 2015 [12]	Thailand	41 *S. dimidiatum* cases; predominantly toenails (96.5%); DLSO (98.2%)	NR	*N. dimidiatum*	KOH 20%; SDA ± cycloheximide; morphology	Retrospective cross-sectional study	Epidemiological characterization
Roy et al., 2015 [33][50]	India	55 M; toenail; diabetes; 3-year history	Oral itraconazole × 24 wk	*N. dimidiatum*	KOH 40%; SDA; cornmeal agar; LPCB; susceptibility testing	Case report	Complete clinical and mycological resolution
Miqueleiz-Zapatero et al., 2017 [4]	Spain	4 reported cases; toenails; 3 cases specified in source	Terbinafine in selected cases; other treatment NR	*N. dimidiatum* / *N. hyalinum*	KOH; SDA + chloramphenicol/gentamicin; LPCB; ITS sequencing	Case series	Poor response reported in nail disease
Leeyaphan et al., 2017 [10]	Thailand	221 fingernails from 1102 patients; *N. dimidiatum* in 2.7% of fingernail onychomycoses	NR	*N. dimidiatum*	KOH 20%; SDA ± cycloheximide; morphology	Retrospective cross-sectional study	Epidemiological characterization
Lavorato et al., 2017 [34]	Brazil	122 confirmed DLSO cases; *N. dimidiatum* isolated in 44.4% of NDM cases	NR	*N. dimidiatum*	KOH + DMSO; SDA/Mycosel; PAS/Grocott histology	Prospective uncontrolled study	Diagnostic yield compared across methods
Di Chiacchio et al., 2017 [35]	Brazil	52 M; toenail; previous hallux melanoma	Mechanical curettage	*N. dimidiatum*	Dermoscopy; KOH 30%; SDA + chloramphenicol	Case report	Clinical resolution after mechanical removal
Veasey et al., 2017 [36]	Brazil	11 *Neoscytalidium* cases among 73 positive cultures from 160 suspected cases; 7 *S. hyalinum*, 4 *N. dimidiatum*	NR	*N. dimidiatum* / *S. hyalinum*	KOH 10% + DMSO; SDA/Mycosel; microculture	Retrospective study, 2003–2015	Epidemiological characterization
Bunyaratavej et al., 2018 [37]	Thailand	9 patients; toenails	*Olea europaea* leaf extract BID × 6 mo + mechanical reduction	*N. dimidiatum*	KOH; repeated fungal culture	Prospective pilot study	Mycological cure: 33% at 6 mo
Alvarado et al., 2018 [38]	Colombia	2 toenail cases among 1221 patients	NR	*N. dimidiatum*	KOH 40%; Sabouraud/Mycosel; ≥ 2 positive cultures; morphology	Cross-sectional descriptive study	Epidemiological characterization
Shokoohi et al., 2020 [8]	Iran	52 F; fingernail	Oral terbinafine + topical ciclopirox	*N. novaehollandiae*	KOH 20%; SDA; ITS sequencing; CLSI susceptibility testing	Case report	Complete clinical and mycological resolution
Razavion et al., 2022[7]	Iran	38 M; toenail	Terbinafine + clobetasol (failed); itraconazole 100 mg/day + amorolfine	*N. dimidiatum*	KOH 15%; SDA; LPCB; ITS sequencing; CLSI susceptibility testing	Case report	Clinical improvement; susceptibility data reported
Lara et al., 2022 [39]	Brazil	74 *Neoscytalidium* cases among 1595 onychomycosis cases	NR	*Neoscytalidium* spp.	KOH 20%; PDA/Sabouraud culture; morphology	Retrospective cross-sectional study	Epidemiological characterization
Alvarez et al., 2022 [40]	Argentina	60 onychomycosis cases	43% had previous topical/systemic treatment	*Neoscytalidium* spp.	KOH; fungal culture	Prospective cross-sectional observational study	78% of previously treated patients showed no clinical improvement
Bersano et al., 2023 [41]	Brazil	31 confirmed onychomycoses among 151 hemodialysis patients; *Neoscytalidium* spp. in 6	NR	*Neoscytalidium* spp.	KOH; Sabouraud ± cycloheximide; macro-/micromorphology	Prospective observational study, 2022–2023	Epidemiological characterization
Chanyac hailer P et al., 2023 [42]	Thailand	184/405 foot/ toenail cases	NR	*N. dimidiatum*	NR	Retrospective cross-sectional study	Outcome not reported
Hakim et al., 2025 [43]	Indonesia	2 cases: toenails (70 F) and fingernails (12 F); concomitant *Candida* spp.	Ketoconazole 2%; fluconazole 150 mg/week + ketoconazole 2%	*N. dimidiatum* + *Candida* spp.	KOH; SDA; LPCB; susceptibility testing	Case report	Case 1 lost to follow-up; case 2 improved at 5 mo
Faver et al., 2025 [44]	Brazil	71 M; toenail; beach/fishing exposure	Mechanical removal	*N. dimidiatum*	Dermoscopy; direct microscopy; Sabouraud/Mycosel; MALDI-TOF	Case report	Clinical resolution without recurrence
Bhalodia et al., 2025 [45]	India	Fingernail	Oral terbinafine + ciclopirox 8%	*N. novaehollandiae*	KOH 40%; SDA/LPCB; arthroconidia; no molecular testing	Case report	Complete recovery within 3 mo
Present case, 2026	Iran	Toenail	Itraconazole + amorolfine 5% lacquer + sertaconazole 2%	*N. dimidiatum*	KOH; repeated SDA cultures; LPCB; ITS sequencing	Case report	Clinical improvement; treatment ongoing

**Abbreviation:** DLSO, distal and lateral subungual onychomycosis; TDO, total dystrophic onychomycosis; SWO, superficial white onychomycosis; PSO, proximal subungual onychomycosis; KOH, potassium hydroxide; SDA, Sabouraud dextrose agar; PDA, potato dextrose agar; LPCB, lactophenol cotton blue; PAS, periodic acid–Schiff; CLSI, Clinical and Laboratory Standards Institute; MIC, minimum inhibitory concentration; NR, not reported; NA, not applicable; NDM, non-dermatophyte mould.

**Nomenclature:** Species names are presented according to current nomenclature where possible. Historical reports using *Scytalidium dimidiatum* or *Scytalidium lignicola* are standardized to *Neoscytalidium dimidiatum* in the table; the original nomenclature should be retained in the reference list and discussed in the text where taxonomically relevant.

## Discussion

*Neoscytalidium dimidiatum* is an uncommon but clinically relevant non-dermatophyte mold (NDM) associated with onychomycosis and other superficial infections. Evidence accumulated over more than four decades indicates that *Neoscytalidium*-associated nail infection occurs across diverse geographic regions and may present with variable clinical patterns and therapeutic responses. Interpretation of this literature is complicated by historical changes in nomenclature, phenotypic overlap among related species, and the potential for environmental molds recovered from nail specimens to represent colonization or contamination. The present case of molecularly confirmed *N. dimidiatum* distal and lateral subungual onychomycosis (DLSO) in an immunocompetent Iranian woman adds to the limited clinical literature on this uncommon cause of NDM onychomycosis.

### Historical emergence and taxonomy

Early reports established the ability of *Neoscytalidium*-related fungi to cause persistent infections involving the skin and nails. Peiris et al. described five patients from the West Indies with *Scytalidium hyalinum* infection involving the palms, soles, toe webs, and toenails; topical imidazole therapy resulted in only partial clinical improvement, and mycological cure was not consistently achieved [13]. Carruthers et al. subsequently reported persistent nail and skin infection caused by an organism identified at the time as *Hendersonula toruloidea* in a patient originally from Fiji, demonstrating both the chronic nature of the infection and the limitations of conventional treatment [14]. Gugnani et al. later described an infection associated with poor response to griseofulvin, ketoconazole, and miconazole, although topical clotrimazole was associated with clinical improvement [15]. Rollman et al. further demonstrated that successful management may require mechanical removal of infected nail tissue when prolonged systemic therapy is unsuccessful [16].

Subsequent reports demonstrated that these infections were not restricted to tropical regions. Summerbell et al. documented 19 cases caused by *N. dimidiatum* and three caused by *S. hyalinum* in Canada, supporting the occurrence of these organisms in temperate settings [17]. Kane et al. reported an autochthonous Canadian case in a patient without travel to a recognized endemic region, further challenging the assumption that infection necessarily reflects recent tropical exposure [18]. Similarly, reports from Australia documented *S. hyalinum* and *N. dimidiatum* in patients without a relevant history of travel [19], [20]. Collectively, these observations suggest that the geographic distribution of *Neoscytalidium* spp. is broader than originally recognized.

Comparison of older reports with contemporary studies requires particular attention to taxonomy. Organisms currently classified as *N. dimidiatum* have historically been reported under several names, including *Hendersonula toruloidea*, *Nattrassia mangiferae*, *Scytalidium dimidiatum*, and *Scytalidium lignicola*. The case series of Lacaz et al. is particularly relevant because it addressed the taxonomy of these organisms and contributed to recognition of the relationship between historical *Scytalidium* designations and *N. dimidiatum* [22]. Consequently, apparent changes in the epidemiology of *Neoscytalidium* over time may partly reflect differences in nomenclature and identification methods rather than genuine changes in geographic distribution.

### Geographic distribution and epidemiology

The published literature demonstrates that *N. dimidiatum* and related *Neoscytalidium* species have been reported in multiple geographic regions. Large clinical studies have shown that these organisms may represent a substantial proportion of NDM-associated nail infections in selected populations. Lacroix et al. identified 332 patients with scytalidiosis in France, including infections caused by *S. hyalinum*, *N. dimidiatum*, and mixed infections [24]. In Colombia, Álvarez et al. identified *N. dimidiatum* among patients with nail lesions [25], while Morales-Cardona et al. subsequently reported 178 confirmed *N. dimidiatum* infections among 317 patients with NDM-associated onychomycosis, with DLSO representing the predominant clinical pattern [32]. In French Guiana, Simonnet et al. identified *N. dimidiatum* in 29 cases, accounting for approximately 71% of NDM isolates associated with toenail disease [28].

Studies from Brazil have also demonstrated a substantial contribution of *Neoscytalidium* spp. to NDM-associated nail disease. Cursi et al. reported 30 cases of *Scytalidium*-associated toenail onychomycosis in a university hospital population, with many patients having received previous topical or systemic treatment [11]. Xavier et al. identified both *S. dimidiatum* and *S. hyalinum* among patients with ungual and cutaneous lesions, with *S. dimidiatum* predominating [27]. Lavorato et al. evaluated diagnostic approaches in patients with clinically suspected toenail onychomycosis and identified *N. dimidiatum* as an important NDM isolate, emphasizing the complementary value of direct microscopy, culture, and histopathology [34]. Lara et al. and Veasey et al. likewise documented *Neoscytalidium* spp. among patients evaluated for onychomycosis [36], [39].

Additional reports from Cameroon, Brazil, Thailand, and other regions further support the broad distribution of these organisms [12], [30], [31], [42], [46]. Importantly, the frequency of *Neoscytalidium* among NDM-associated nail infections varies considerably between studies. Such differences may reflect geographic variation, patient selection, diagnostic methodology, criteria used to define NDM infection, and the relative contribution of dermatophytes and other NDMs within individual populations. Therefore, isolation frequency from one geographic region should not necessarily be interpreted as representative of the global epidemiology.

The organism has also been reported in specific patient populations. Alvarado et al. detected *N. dimidiatum* among fungal infections in children and adolescents in Colombia [38], while studies from Argentina and Brazil documented *Neoscytalidium*-associated onychomycosis among previously treated patients and individuals undergoing hemodialysis, respectively [40], [41]. These observations indicate that *Neoscytalidium* infection is not restricted to a particular age group or host population.

### Clinical spectrum and predisposing factors

Across published reports, toenail involvement—particularly DLSO of the great toenail—is the most frequently described clinical presentation. Nevertheless, fingernail infection, palmoplantar involvement, interdigital disease, and concomitant cutaneous and ungual infection have also been reported [13], [14], [16], [17], [19], [20]. The present case is consistent with the predominant clinical pattern, with isolated toenail DLSO in an immunocompetent patient and no clinically apparent concomitant cutaneous infection.

Local trauma and environmental exposure may contribute to the development of infection. Occupational exposure was proposed as a potential risk factor in two Indian green tea leaf pluckers with thumbnail infection caused by *N. dimidiatum* [47]. Infection has also been documented among Colombian university students [26], while Faver et al. described a patient with frequent beach exposure and recreational fishing, suggesting repeated environmental contact as a possible source of exposure [44]. In the present case, the history of trauma to the affected toenail approximately one year before presentation may have contributed to disruption of the nail barrier and facilitated subsequent fungal establishment. However, a causal relationship cannot be established from a single case.

Although nail infection can occur in otherwise healthy individuals, immunosuppression may increase the risk of more extensive or invasive disease. Lacaz et al. reported *Neoscytalidium* infection in an HIV-positive patient [22], while Garinet et al. described invasive cutaneous infection in renal transplant recipients [48]. These observations highlight the broader pathogenic potential of the genus. Nevertheless, the occurrence of isolated nail disease in immunocompetent individuals, including the present patient, indicates that severe host immunosuppression is not a prerequisite for *N. dimidiatum* onychomycosis.

Mixed fungal infections represent another important consideration. Peiris et al. described mixed infections involving *S. hyalinum* and dermatophytes as well as a case involving *N. dimidiatum* [13]. Lacroix et al. similarly reported mixed infections with dermatophytes [24], while Hakim et al. documented *N. dimidiatum* together with *Candida albicans* and *Candida glabrata* in separate cases of superficial black onychomycosis [43]. Thus, isolation of *N. dimidiatum* from a nail specimen should be interpreted in the context of the complete clinical and mycological findings rather than automatically considered the sole etiological agent.

### Diagnostic challenges and molecular identification

Accurate diagnosis of *N. dimidiatum* onychomycosis requires correlation of clinical findings with direct microscopy and fungal culture. Historically, identification was based largely on colony morphology and microscopic characteristics. However, these features may overlap with those of other melanized fungi and closely related *Neoscytalidium* species, particularly *N. novaehollandiae*. In addition, environmental distribution of these molds creates an inherent concern regarding contamination or transient colonization of nail specimens.

Because non-dermatophyte molds may be environmental contaminants or transient colonizers of the nail, their isolation from a single nail specimen does not by itself establish causality. Several complementary criteria have therefore been proposed for diagnosing NDM-associated onychomycosis, including (i) demonstration of fungal hyphae by direct microscopy, particularly KOH preparation; (ii) isolation of the same NDM in culture; (iii) repeated isolation of the same organism from independently collected nail specimens; (iv) a significant inoculum or colony burden when quantitative assessment is available; (v) absence of a dermatophyte in culture; and (vi) histopathological demonstration of fungal elements within the nail. Previous reviews have suggested that fulfillment of at least three of these criteria, including positive direct microscopy and culture, provides stronger evidence against contamination. Repeated recovery of the same mold from sequential specimens is particularly useful when distinguishing persistent infection from incidental environmental contamination. Histopathology may provide additional evidence of fungal invasion, while molecular identification can resolve species-level uncertainty but should be interpreted together with clinical and mycological findings because detection of fungal DNA alone does not necessarily establish viability or pathogenicity.

The distinction between true infection, colonization, and contamination is particularly important in NDM-associated nail disease. A single positive culture may reflect environmental contamination of the nail surface or laboratory contamination, whereas persistent recovery of the same organism from sequential specimens increases the likelihood of clinically significant infection. Transient or secondary colonization may also occur, particularly when the nail has been previously traumatized or affected by another nail disorder. Therefore, the interpretation of an NDM isolate should be based on the concordance of the clinical phenotype, direct microscopy, culture results, and, when available, histopathology. Demonstration of fungal elements directly within the nail, repeated isolation of the same mold, absence of a dermatophyte, and evidence of tissue invasion provide progressively stronger support for true infection. Conversely, isolation of an NDM in the absence of fungal elements on direct examination, without reproducibility on repeat culture or other evidence of invasion, should be interpreted cautiously and may represent contamination or colonization rather than the primary cause of nail dystrophy. Molecular identification is valuable for confirming the identity of the recovered organism but does not, by itself, establish that the organism is the etiologic agent.

Morales-Cardona et al. used repeated recovery in combination with positive direct microscopy to support the diagnosis of NDM-associated onychomycosis [32], while similar approaches have been reported in studies from Thailand and Colombia [12], [38]. Histopathology may provide additional evidence when culture findings are equivocal. Elewski demonstrated the diagnostic value of nail histopathology in a case with initially confusing culture results, while Lavorato et al. emphasized that direct microscopy, culture, and histopathology provide complementary diagnostic information [21], [34].

Molecular identification has become increasingly useful for resolving species-level diagnostic uncertainty. Miqueleiz-Zapatero et al. used ITS sequencing to confirm *Neoscytalidium* species in clinical cases from Spain [4]. Shokoohi et al. subsequently reported a molecularly confirmed fingernail infection caused by *N. novaehollandiae* in Iran, demonstrating that species diversity within the genus extends beyond *N. dimidiatum* [8]. Importantly, Razavyoon et al. reported *N. dimidiatum* onychomycosis in an Iranian patient and confirmed the isolate by ITS sequencing, together with antifungal susceptibility testing [7]. Thus, the present case should not be considered the first report of *N. dimidiatum* onychomycosis in Iran; rather, it represents an additional molecularly confirmed Iranian case with a different clinical and demographic profile.

The present case further illustrates the value of integrating conventional and molecular methods. Direct microscopy demonstrated fungal elements, culture yielded a rapidly growing melanized mold with compatible morphology, and ITS sequencing demonstrated greater than 99% identity with *N. dimidiatum*. Importantly, repeated sampling on separate occasions, together with positive microscopy and compatible culture findings, strengthened the evidence that the isolate represented true nail infection rather than incidental contamination. The concordance of these findings provides a robust basis for species-level diagnosis.

### Antifungal susceptibility and therapeutic management

Treatment outcomes reported for *N. dimidiatum* onychomycosis have been heterogeneous. Early reports frequently described poor or incomplete responses to conventional antifungal therapy. Peiris et al. reported limited benefit from prolonged griseofulvin and incomplete mycological responses to topical imidazoles [13]. Carruthers et al. similarly documented persistent infection despite treatment [14], while Gugnani et al. reported poor activity of griseofulvin, ketoconazole, and miconazole, with clinical improvement following clotrimazole therapy [15]. Rollman et al. demonstrated successful management after mechanical nail avulsion and curettage combined with topical ciclopiroxolamine when prolonged griseofulvin therapy had failed [16].

Subsequent reports have provided further evidence of variable antifungal susceptibility. Elewski documented resistance to several systemic agents, including griseofulvin, ketoconazole, fluconazole, itraconazole, and terbinafine [21]. Arrese et al. likewise described persistent infection despite treatment with fluconazole, itraconazole, or terbinafine [23]. These findings suggest that therapeutic failure may reflect organism-specific susceptibility patterns, although differences in nail penetration, disease duration, adherence, and treatment duration must also be considered.

Conversely, successful outcomes have been reported with several therapeutic strategies. Kane et al. described successful treatment using topical boric acid in mineral oil [18]. Pontarelli et al. reported successful management with systemic antifungal therapy combined with topical amorolfine in one of two cases [49]. Roy et al. documented complete clinical and mycological resolution after 24 weeks of oral itraconazole in a diabetic patient [33]. Razavyoon et al. reported clinical improvement in an Iranian patient treated with itraconazole and amorolfine nail lacquer, further supporting the potential utility of combined systemic and topical therapy [7].

In the present case, antifungal susceptibility testing was not performed for the *N. dimidiatum* isolate because it had not been requested during the initial diagnostic evaluation, and antifungal therapy was initiated promptly after the initial mycological results became available. Consequently, the selected treatment regimen was not susceptibility-guided, and the observed clinical improvement cannot be directly linked to an isolate-specific in vitro susceptibility profile.

Susceptibility-guided treatment may be particularly useful in recalcitrant disease. Spriet et al. reported successful eradication of combined ungual and cutaneous *N. dimidiatum* infection after failure of multiple antifungal agents, using voriconazole guided by susceptibility testing and therapeutic drug monitoring [29]. Although such an approach may be appropriate in selected refractory infections, the systemic use of voriconazole requires careful consideration because of potential adverse effects, drug interactions, and the need for monitoring.

Mechanical reduction of infected nail tissue may also contribute to successful treatment. Nail avulsion, curettage, and other forms of mechanical reduction have been associated with favorable outcomes in several reports [16], [18], [29]. Faver et al. reported clinical resolution following mechanical removal alone, while Di Chiacchio et al. described successful mechanical curettage in a case in which the differential diagnosis included subungual melanoma [35], [44]. These observations suggest that reduction of fungal burden and improvement of topical drug penetration may be useful adjuncts, particularly in markedly dystrophic or hyperkeratotic nails.

Other adjunctive approaches have been investigated. Bunyaratavej et al. reported a pilot study combining fermented olive leaf extract with mechanical nail reduction, with clinical improvement but a mycological cure rate of approximately one-third at six months [37]. However, the limited number of controlled studies and substantial heterogeneity among treatment regimens prevent meaningful comparison of efficacy across the published literature.

In the present case, treatment consisted of oral itraconazole combined with amorolfine 5% nail lacquer and topical sertaconazole 2%. Marked clinical improvement was observed after three months. Because treatment was ongoing at the time of reporting and long-term mycological follow-up was not yet available, this early response should not be interpreted as definitive clinical or mycological cure. Nevertheless, the observed improvement is consistent with the favorable responses reported with combined systemic and topical therapy in selected cases.

### Species diversity and taxonomic considerations

Although *N. dimidiatum* remains the predominant *Neoscytalidium* species reported in the nail literature, recognition of *N. novaehollandiae* as a human nail pathogen highlights the importance of species-level identification. Shokoohi et al. reported a molecularly confirmed fingernail infection caused by *N. novaehollandiae* in Iran, with clinical resolution following terbinafine and ciclopirox therapy [8]. Bhalodia et al. subsequently reported fingernail onychomycosis caused by the same species in India [45]. These observations indicate that the clinical spectrum of *Neoscytalidium*-associated onychomycosis is broader than suggested by studies focused exclusively on *N. dimidiatum*.

Historical taxonomic changes further complicate comparison among studies. Many earlier reports were based on morphology and used nomenclature that does not correspond directly to current species concepts. Accordingly, epidemiological estimates derived from older literature should be interpreted cautiously. Future studies should preferentially use validated molecular or proteomic approaches for species-level identification, particularly when isolates are unusual, recurrent, or associated with treatment failure [50], [51].

### Strengths and limitations

A major strength of the present report is the integration of detailed clinical assessment with repeated mycological sampling, direct microscopy, culture, and molecular identification. The case also contributes to the relatively limited literature on *N. dimidiatum* onychomycosis from Iran by documenting an immunocompetent female patient with a clinically defined DLSO pattern and a clear history of preceding nail trauma.

An additional limitation of the present case is that antifungal susceptibility testing could not be performed for the *N. dimidiatum* isolate. Treatment was initiated promptly after the initial mycological results became available, and susceptibility testing had not been requested during the initial diagnostic evaluation. Consequently, the choice of antifungal therapy was based on the available clinical literature and previously reported treatment experience rather than isolate-specific susceptibility data. This limits the ability to correlate the observed clinical response with the in vitro susceptibility profile and precludes any inference regarding the susceptibility or resistance of this particular isolate. Given the variable antifungal susceptibility patterns reported for *N. dimidiatum*, susceptibility testing may be particularly informative in persistent or refractory NDM infections [52], [53], [54], [55], [56].

The literature review provides additional context by demonstrating that *Neoscytalidium*-associated nail infection has been reported across multiple geographic regions and in diverse patient populations. However, the available evidence is dominated by case reports, case series, and retrospective observational studies. Diagnostic criteria are inconsistent, historical species identification was frequently morphology-based, antifungal susceptibility testing has not been uniformly performed, and treatment regimens vary considerably. Furthermore, long-term mycological follow-up is often insufficiently reported. These limitations preclude direct comparison of treatment efficacy and prevent the establishment of a standardized organism-specific therapeutic regimen.

**In conclusion**, evidence accumulated over more than four decades indicates that *Neoscytalidium dimidiatum* is an established but uncommon cause of non-dermatophyte mold onychomycosis and can affect both immunocompetent and immunocompromised individuals. Although historically associated with tropical and subtropical regions, reports of autochthonous infections from temperate settings indicate that its geographic and ecological distribution is broader than previously recognized. Diagnosis remains challenging because of morphological overlap with other melanized molds, historical changes in taxonomy, and the potential coexistence of *N. dimidiatum* with dermatophytes or yeasts. Accordingly, careful correlation of clinical findings with direct microscopy and culture, supplemented by repeated sampling, histopathology, or molecular identification when appropriate, is important for establishing clinical significance and accurate species-level diagnosis. Therapeutic management remains heterogeneous, reflecting variable antifungal susceptibility and the limited evidence available to guide organism-specific treatment. Individualized approaches incorporating systemic and topical antifungal therapy and, when appropriate, mechanical nail reduction may therefore be required, particularly in persistent or recalcitrant infections. The present molecularly confirmed Iranian case adds to the limited literature on *N. dimidiatum* onychomycosis by documenting isolated DLSO in an immunocompetent woman and demonstrating concordant clinical, mycological, and molecular findings. These observations reinforce the importance of considering *N. dimidiatum* in atypical or persistent NDM-associated onychomycosis and highlight the value of accurate laboratory identification for appropriate clinical management.

## CRediT authorship contribution statement

**Fuad Haghighat:** Writing – review & editing, Writing – original draft, Investigation, Conceptualization. **Seyed Jamal Hashemi:** Supervision, Project administration, Methodology, Conceptualization. **Shahram Mahmoudi:** Validation, Software, Investigation, Data curation. **Abbas Rahimi Foroushani:** Software, Methodology, Formal analysis. **Kimia Kamali Sarvestani:** Validation, Software, Investigation. **Mohsen Gerami Shoar:** Validation, Investigation, Formal analysis. **Roshanak Daie Ghazvini:** Validation, Supervision, Methodology. **Leila Hossein pour:** Investigation, Formal analysis, Conceptualization. **Mahsa Naieem:** Writing – original draft, Visualization, Investigation, Conceptualization. **Hasti Kamali Sarvestani:** Supervision, Project administration, Methodology, Conceptualization.

## Ethics declaration

Written informed consent to take part in the study and to publish the article has been obtained from all participants or their legal representatives. The privacy rights of participants have been observed.

This study included organ or tissue donors. This study includes human biological material and consent was obtained by donors, or their next of kin or legal representatives, for use in this study and for publication of the article. The samples used in this research were not sourced from executed prisoners or prisoners of conscience.

This study was performed in compliance with relevant laws, regulatory frameworks and guidelines where the research took place. This study was approved by the Tehran University of Medical Sciences (Approval No. IR.TUMS.SPH.REC.1405.017).

This research follows the CARE guidelines and the CARE checklist.

## Declaration of Competing Interest

The authors declare that they have no known competing financial interests or personal relationships that could have appeared to influence the work reported in this paper.
